# Predictive Biomarkers in Laser Dermatology: Toward Personalized Energy‐Based Treatments

**DOI:** 10.1111/jocd.70991

**Published:** 2026-06-15

**Authors:** Diala Haykal, Alexandre de Almeida Filippo, Gustavo Robertson Filippo, Marco Rocha, Ashraf Badawi

**Affiliations:** ^1^ Centre Médical Laser Palaiseau, Private Practice Palaiseau France; ^2^ Laser and Technology Department Institute of Dermatology Ruben David Azulay Rio de Janeiro Brazil; ^3^ Instituto De Dermatologia Rubem David Azulay Rio de Janeiro Brazil; ^4^ Federal University of São Paulo São Paulo Brazil; ^5^ National Institute of Laser Enhanced Sciences, Cairo University Giza Egypt; ^6^ Department of Dermatology and Allergology University of Szeged Szeged Hungary

## Abstract

**Background:**

Despite major technological advances in laser and energy‐based devices, adverse events such as post‐inflammatory hyperpigmentation (PIH), prolonged erythema, delayed wound healing, and dyschromia remain significant clinical challenges. Current risk assessment largely relies on Fitzpatrick Skin Type classification and clinical judgment, which inadequately capture the biological heterogeneity influencing individual treatment responses.

**Objective:**

To explore emerging biological biomarkers and predictive technologies that may improve individualized risk stratification, treatment planning, and complication prevention in laser dermatology, thereby supporting the transition toward precision medicine.

**Methods:**

This perspective article reviews current evidence regarding biological determinants of laser‐induced adverse events, including skin barrier integrity, inflammatory activity, pigmentary response pathways, oxidative stress, microbiome composition, and biological aging. Potential applications of minimally invasive biomarker assessment and artificial intelligence (AI)‐driven predictive modeling are discussed within the context of personalized energy‐based treatments.

**Results:**

Emerging biomarkers such as transepidermal water loss (TEWL), epidermal cytokine profiles obtained through tape stripping, melanogenic gene expression signatures, oxidative stress markers, microbiome‐associated parameters, and indicators of biological age may provide clinically meaningful information beyond conventional phototype assessment. These markers have the potential to identify patients at increased risk of adverse outcomes, enabling tailored treatment parameters, optimized preconditioning strategies, and enhanced posttreatment monitoring. Furthermore, AI‐based platforms may integrate multimodal biological, imaging, and clinical data to generate individualized safety predictions and support real‐time treatment optimization.

**Conclusions:**

A biology‐centric approach to laser dermatology may complement existing safety frameworks by incorporating objective molecular and biophysical markers into clinical decision‐making. Although many proposed biomarkers remain investigational, their future integration, particularly when combined with AI‐driven analytics, may improve treatment predictability, reduce complications, and advance equitable, personalized care across diverse patient populations. Further validation studies are needed to establish clinical feasibility, reproducibility, and cost‐effectiveness before routine implementation.

## 1 Introduction

Laser dermatology has undergone a remarkable technological evolution over the past decade, with devices now offering unprecedented precision, uniform energy delivery, advanced pulse shaping, and increasingly sophisticated cooling systems. While these innovations have substantially improved efficacy and tolerability, adverse events such as post‐inflammatory hyperpigmentation, prolonged erythema, delayed re‐epithelialization, and dyschromia remain clinically relevant challenges [1].

As dermatology progressively transitions toward precision medicine and biologically personalized care, there is increasing recognition that conventional phenotype‐based assessments alone may be insufficient to predict individual responses to energy‐based treatments. This evolving landscape has generated growing interest in biomarker‐driven and biology‐centric approaches aimed at improving personalization, predictability, and complication prevention in laser dermatology.

This perspective article aims to contribute to the evolving discussion surrounding precision medicine and personalized risk prediction in energy‐based dermatology. While several proposed biomarkers and technologies remain investigational or primarily research‐based, their future integration may help refine individualized treatment planning and improve clinical outcomes across diverse patient populations.

## 2 Why Risk Stratification Requires a New Predictive Model

Traditional safety assessments in laser dermatology rely heavily on the Fitzpatrick phototype and broad clinical impressions such as “sensitive,” “reactive,” or “inflammatory‐prone” skin. While these heuristics offer a convenient framework, they provide limited insight into the underlying molecular and biophysical mechanisms that dictate how skin responds to thermal injury. Phototype does not capture barrier integrity, cytokine milieu, oxidative status, melanin density gradients, or transcriptomic signatures, all of which critically influence healing trajectories and complication risk. Thus, the use of the Fitzpatrick scale is important in helping the analysis of patient skin, but other factors must be considered to have more accuracy in laser treatment [2].

Emerging evidence from barrier science, inflammation research, pigment biology, and transcriptomic profiling now shows that objective biomarkers could enhance predictive capability. Metrics such as transepidermal water loss (TEWL), natural moisturizing factor (NMF) ratios, baseline inflammatory cytokines, melanin dispersion patterns, or gene expression signatures associated with wound healing can reveal vulnerabilities invisible to the naked eye (Table 1).

**TABLE 1:** Traditional laser safety assessment vs. Biology‐Centric predictive model.

Aspect of safety evaluation	Traditional approach	Biology‐centric predictive model
**Primary Risk Stratification Tool**	Fitzpatrick phototype; subjective assessment of sensitivity	Objective molecular and biophysical markers (TEWL, cytokines, melanin maps, oxidative load)
**Ability to Predict PIH**	Low; based on phenotype heuristics	High; integrates inflammatory status, pigment gene expression, and baseline melanocyte reactivity
**Assessment of Barrier Function**	Clinical impression (dryness, erythema)	Quantitative barrier metrics (TEWL, lipidomics, NMF)
**Evaluation of Inflammatory Proneness**	Patient history or visible erythema	Cytokine signatures, transcriptomic inflammation profiles
**Prediction of Healing Trajectory**	Based on clinician experience	Biological age indicators, mitochondrial function, wound‐healing gene expression
**Personalization of Laser Parameters**	Broad parameter adjustments (energy, fluence, cooling)	Precision‐guided parameters tailored to molecular readiness
**Equity Across Skin Tones**	Limited; higher unpredictability in dark phototypes	Increased safety through individualized biology‐informed risk prediction
**Overall Limitations**	Oversimplified, phenotype‐based, high inter‐operator variability	Data‐driven, reproducible, measurable, aligned with precision medicine

Recognizing these biological determinants has prompted a conceptual shift: Moving from device‐centered predictive assessment, where risk is primarily managed by adjusting energy, pulse duration, or wavelength, to a biology‐centric model that stratifies risk based on the patient's molecular readiness for injury and repair. Such an approach aligns laser dermatology with broader trends in precision medicine and positions the field to reduce disparities, improve predictability, and enhance treatment outcomes across diverse populations.

## 3 Limitations of Current Predictive Tools

Patients classified within the same Fitzpatrick Skin Type (FST) may nonetheless exhibit markedly different healing trajectories and risks of PIH [3, 4].

This variability reflects the inherent limitations of the Fitzpatrick scale, which remains a subjective tool reliant on patient self‐assessment and physician clinical judgment, both of which may be imprecise. Moreover, the Fitzpatrick classification evaluates acute ultraviolet sensitivity, a biological response that differs fundamentally from the skin's reaction to controlled, high‐intensity thermal stimuli such as laser energy [5]. Although patient history and clinical examination are essential components of pre‐procedural assessment, they are inherently limited by variability in accuracy, completeness, and interpretability. Even meticulous clinical evaluations often fail to detect subclinical inflammation, occult pigmentary disorders (such as melasma or rosacea), the use of photosensitizing medications, compromised skin barrier function, oxidative stress burden, microbiome imbalance, or intrinsic healing capacity, factors increasingly recognized as critical determinants of outcomes following laser‐based procedures [6, 7].

As a result, inadequate or oversimplified phototype assessment may predispose patients to adverse events, including:

1. Burns: Due to excessive energy absorption, leading to pain, bullae formation, and increased risk of secondary infection.

2. Dyschromia: Including post‐inflammatory hyperpigmentation (particularly in FST IV–VI due to excessive thermal load) or hypopigmentation resulting from melanocyte destruction within the treated area.

3. Pathologic scarring: Such as hypertrophic, hypotrophic, or keloid scars, with responses varying according to anatomical site and underlying genetic susceptibility [4].

In parallel, the relationship between FST classification and ethnic or racial background warrants careful consideration. Ethnic and racial classifications, based on shared ancestry, geographic origin, physical traits, and cultural identity (e.g., White, Black, Asian, Hispanic/Latino), would correlate with broader patterns of skin biology but should not be used as proxies for phototype. While darker FSTs (IV–VI) are statistically more prevalent within certain ethnic groups and are associated with higher risks of laser‐induced burns or dyspigmentation, substantial intragroup variability exists. For example, individuals of South Asian or Hispanic heritage may span a wide range of phototypes (III–VI), underscoring the limitations of ethnicity‐based assumptions.

Recognizing the distinction between phototype and ethnic background is essential for equitable and safe aesthetic care. An individualized, biologically informed assessment that integrates phototype, clinical history, skin biology, and contextual factors is therefore critical to optimizing outcomes and minimizing complications across diverse patient populations [7].

## 4 Mechanistic Basis of Laser Adverse Events

Laser‐induced complications are biologically multifactorial and reflect the interaction of inflammatory, barrier, oxidative, and pigmentary pathways. Inflammatory reactivity plays a pivotal role: Individuals differ significantly in baseline cytokine activity, with heightened inflammatory states predisposing to prolonged erythema and PIH. Barrier dysfunction further exacerbates vulnerability. Increased TEWL and reduced lipid content impair the skin's ability to tolerate thermal injury and delay re‐epithelialization, especially after fractional resurfacing.

Oxidative stress represents a third axis of vulnerability. Elevated reactive oxygen species (ROS) amplify both inflammation and melanogenesis, thereby increasing the likelihood of pigmentary sequelae. Finally, melanocyte sensitization, reflected not only in melanin quantity but also in gene expression patterns, determines the intensity of pigmentary responses. Together, these mechanisms underline the need for biologically anchored predictors that reveal how each individual's skin is likely to behave under energy‐based stimulation [8].

### 4.1 Strategy and Additional Considerations for Phototype Evaluation in Different Clinical Contexts

To minimize risks and side effects, some clinical assessments, prior history, and practical care can be performed:

#### 4.1.1 Detailed Medical History [7, 9, 10]

- History of Sun Exposure: Previous sunburns, tanning ability.
- History of PIH: Inquire about the development of dark spots after previous trauma, inflammation, or cosmetic procedures.
- Family and Ethnic History: Genetic predisposition and ancestry can influence skin response.
- Medications: Use of isotretinoin, tetracyclines, and anti‐inflammatory drugs, among others, can increase photosensitivity or alter healing.
- Dermatological Conditions: Melasma, vitiligo, atopic dermatitis, and recurrent herpes labialis may worsen after treatment.

#### 4.1.2 Thorough Physical Examination [7, 9, 10, 11]

- Visual Assessment of Pigmentation: Observe skin color in areas not exposed to the sun, such as the gluteal region, for a more precise assessment of the constitutive pigment.
- Presence of Pre‐existing Lesions: Identify pigmented lesions, scars, or areas of inflammation that may be exacerbated by the laser.
- Spot Test: In cases of doubt or in higher phototypes, performing a spot test on a discreet area (e.g., behind the ear) is essential. It allows observation of the skin's response to the laser with specific parameters before treating a larger area. The assessment should be done after 4–6 weeks to observe the development of PIH [7].

#### 4.1.3 Auxiliary Technologies [4, 9, 10, 11]

- Cutaneous Spectrophotometry: Objective measurement of melanin and erythema index, offering quantitative data for a more precise assessment. Studies show that reflectance spectrophotometry can objectively differentiate between phototypes and correlate better with UV sensitivity than subjective classification.
- Dermatoscopic or Polarized Light Imaging: Can assist in visualizing pigmentation patterns and identifying areas at risk.
- Gender and Age Analysis: Factors that influence skin thickness, hair follicle density, and recovery capacity.

#### 4.1.4 Practical Predictive Assessment Tips [7, 12]

- Educational and continuing training: Increase the knowledge of which chromophore will be targeted, which depth will be achieved, and how to apply the technology correctly (passes, fluence, and spacing between laser shots), individualizing each patient's protocol indication.
- Proper Cooling: The use of effective cooling systems (contact, cold air, cryogen) is crucial to protect the epidermis and minimize the risk of burns and PIH, especially in darker skin tones.
- Skin Preconditioning: In higher phototypes, the use of depigmenting agents (e.g., hydroquinone, retinoids) and sunscreen for 2–4 weeks before the procedure can reduce the risk of PIH.

## 5 Emerging Biological Predictors of Laser Complications

### 5.1 Barrier Function Biomarkers

TEWL has emerged as one of the most clinically accessible and informative biomarkers of pre‐treatment risk. Elevated TEWL reflects impaired barrier integrity and predicts greater susceptibility to irritation, slower recovery after resurfacing, and a higher likelihood of pigmentary alterations. Complementary markers such as corneocyte fragility and ceramide depletion further characterize the degree of barrier vulnerability. These measurements capture the skin's functional resilience in ways that visual inspection simply cannot [13].

### 5.2 Inflammatory Cytokine Profiling via Tape Stripping

Tape stripping allows minimally invasive sampling of epidermal cytokines, including IL‐1α, IL‐6, IL‐8, and TNF‐α. Elevated concentrations of these mediators reflect a hyperreactive cutaneous environment and correlate with exaggerated erythema, prolonged inflammatory response, and increased PIH risk. Importantly, tape stripping is rapid and easily integrated into clinical workflow, making it one of the most promising tools for biological risk assessment [14].

### 5.3 Pigmentary Response Signatures

Melanocyte behavior is largely governed by gene expression, with markers such as MITF, TYR, DCT, and SOX10 providing insight into an individual's pigmentary reactivity. Even in patients sharing identical phototypes, upregulation of melanogenic pathways predisposes to more intense and persistent PIH. Non‐invasive imaging modalities, including melanin index and diffuse reflectance spectroscopy, offer complementary, quantitative assessments of pigment distribution, enabling a more nuanced approach to predicting pigmentary complications [15].

### 5.4 Oxidative Stress Indicators

Biomarkers such as malondialdehyde (MDA) and 8‐hydroxy‐2′‐deoxyguanosine (8‐OHdG) reflect heightened oxidative burden and correlate with delayed wound healing and dyschromia. Oxidative stress influences fibroblast function, inflammatory signaling, and melanocyte activation. Its measurement provides a valuable window into the skin's reparative capacity following fractional or ablative laser treatments [16].

### 5.5 Microbiome‐Based Predictors

The skin microbiome operates as a functional extension of epidermal barrier integrity and immune regulation, and its relevance to personalized laser dermatology is best understood through its interaction with quantifiable biological markers. Barrier impairment, objectively reflected by elevated TEWL, altered lipid composition, or reduced NMF, has been shown to destabilize resident microbial communities, promoting dysbiosis and heightened innate immune signaling. This dysbiotic shift is increasingly associated with measurable increases in epidermal pro‐inflammatory cytokines, including IL‐1α and IL‐8, detectable through minimally invasive techniques such as tape stripping.

Microbiome imbalance, characterized by reduced commensal diversity or enrichment of pro‐inflammatory taxa, has been linked to delayed re‐epithelialization, exaggerated inflammatory cascades, and impaired post‐injury repair. Importantly, microbial‐derived signals can further exacerbate barrier dysfunction through antimicrobial peptide dysregulation and cytokine amplification, establishing a self‐reinforcing inflammatory loop that may remain clinically unapparent. When laser‐induced thermal injury is applied to this biologically primed environment, the result may be disproportionate cytokine release, prolonged erythema, delayed healing, and an increased risk of pigmentary or textural adverse events.

Although routine microbiome profiling is still emerging, its integration with established biomarkers, such as TEWL, epidermal cytokine signatures, and oxidative stress markers, offers a compelling framework for refining pre‐treatment risk stratification. Rather than serving as an isolated parameter, microbiome status may act as a contextual biomarker reflecting underlying barrier competence and inflammatory readiness, thereby contributing meaningfully to predictive models of laser tolerance and healing quality [17, 18].

### 5.6 Senescence and Biological Age

Chronological age is an imprecise predictor of healing. Biological age, reflected in fibroblast senescence burden, extracellular matrix exhaustion, and epigenetic aging, more accurately reflects cutaneous resilience. Skin rich in senescent cells exhibits delayed collagen remodeling and prolonged downtime. Simple assessments of elasticity, barrier function, or epigenetic age could meaningfully enhance pre‐treatment stratification [19, 20].

### 5.7 Toward a Clinically Relevant, Biomarker‐Driven Safety Model

Translating these insights into practice requires an integrated approach. Incorporating TEWL measurement, melanin quantification, and a small cytokine tape‐strip panel into routine evaluation could yield a clinically meaningful biological risk profile. Patients characterized by elevated TEWL or heightened inflammatory signatures may benefit from more conservative parameters, increased spacing between sessions, or pre‐conditioning regimens involving barrier repair, anti‐inflammatory agents, or antioxidants. Conversely, individuals demonstrating favorable biological markers may safely tolerate more aggressive laser settings with reduced downtime [4, 21].

Post‐treatment monitoring could also evolve. Early shifts in cytokine levels or barrier measurements may identify aberrant healing patterns before they appear clinically, enabling timely intervention and reducing the likelihood of long‐term sequelae.

### 5.8 The Future: Integrating AI and Molecular Data

Artificial intelligence represents a natural extension of this biomarker‐driven model, serving as the analytical engine capable of synthesizing the immense complexity of cutaneous biology. Machine learning systems can integrate multimodal datasets, clinical photography, multispectral imaging, reflectance confocal microscopy, spectroscopic readouts, cytokine signatures, melanin and hemoglobin maps, biomechanical properties, microbiome profiles, and patient history to generate individualized safety predictions and parameter recommendations with unprecedented precision [22].

Beyond classification, AI can model biological responses over time, forecasting the skin's dynamic reaction to thermal injury, collagen remodeling, pigment release, or inflammatory cascades. Neural networks trained on longitudinal datasets could identify subtle pre‐treatment vulnerabilities, such as impaired barrier recovery rates, heightened oxidative stress, or pro‐inflammatory epigenetic states, before they translate into clinical complications.

This convergence of AI with biological markers has the potential to standardize safety across practitioners, democratize expertise globally, and reduce variability driven by operator experience. As datasets grow more diverse, AI systems can help counteract long‐standing disparities in laser outcomes for patients with darker phototypes, unique genetic backgrounds, or inflammatory‐prone conditions. Ultimately, AI‐enabled molecular stratification can transform energy‐based treatments from experience‐driven procedures into predictable, personalized therapeutic interventions [23, 24].

AI is a promising tool for the revolution of reality, introducing an unprecedented level of precision, personalization, and efficiency.

#### 5.8.1 Potential Benefits

The application of AI in laser therapy can generate significant advances:

• Diagnostic and Indicative Accuracy: AI algorithms, if trained on vast databases of clinical images and patient histories, can assist in identifying conditions that benefit from laser therapy, surpassing human capacity in detecting subtle patterns and stratifying risk [25, 26].

• Treatment Personalization: By analyzing individual patient data (including phototype, medical history, physiological characteristics of the skin/tissue, and responses to previous treatments), AI can recommend the most appropriate laser protocol, promoting truly personalized medicine [26].

• Parameter Optimization: AI may continuously refine laser parameters (power, frequency, pulse duration, laser type) in real time or pre‐procedure, seeking to maximize therapeutic efficacy and minimize the risks of adverse events such as burns or dyschromia [25].

• Predictive Outcome Analysis: AI‐based predictive models can estimate the probability of treatment success and the risk of complications, allowing physicians and patients to make more informed decisions [25, 27].

### 5.9 Challenges and Limitations

Despite its vast potential, the implementation of AI in laser therapy faces considerable obstacles:

• Need for Large Datasets: Training robust AI models requires large volumes of high‐quality, diverse, and well‐annotated clinical data, which is challenging due to data fragmentation and patient privacy [25, 26].

• Interpretability (“Black Box”): Many AI models, especially deep neural networks, operate as “black boxes,” making it difficult to understand how they arrive at their conclusions. In medicine, explainability (XAI) is crucial for clinical trust and accountability [28].

• Clinical Validation and Regulation: Rigorous validation in clinical trials is essential to prove the safety and efficacy of AI solutions. Furthermore, the regulatory framework for AI‐based medical devices is still evolving [23].

• Implementation and Infrastructure Costs: Acquiring specialized hardware, software, and maintaining data infrastructures represents a significant investment.

• Data Security and Privacy: Handling sensitive patient data requires robust cybersecurity protocols and compliance with regulations such as the LGPD/GDPR [25, 26].

• Dependence on Specific Hardware and Technical Expertise: AI integration requires compatible equipment and professionals with expertise in AI and data science, in addition to medical knowledge [25].

## 6 Future Directions

The future of AI in laser therapy points to:

• Real‐Time AI: Development of systems that dynamically adjust laser parameters during the procedure, based on instant feedback from the laser‐tissue interaction [25].

• Integration with Medical Imaging Systems: Fusion of AI data with multimodal images (ultrasound, OCT, confocal microscopy) for a deeper understanding of tissue anatomy and physiology. Explainable AI (XAI) Systems: Focus on developing models that not only provide recommendations but also explain the reasoning behind them, increasing confidence and clinical acceptance [28].

• Automation and Standardization of Protocols: AI systems can standardize parameter selection and procedure execution, reducing variability between operators and ensuring consistent treatment quality. It can also personalize the parameters according to the identified usage pattern of each physician, adapting to the intensity and experience of each to generate results and post‐procedure care according to the satisfaction and safety of each physician (more conservative or advanced) [25].

Continuous Learning Platforms: Systems that learn and improve with each new treatment performed, refining their models and recommendations over time [29].

## 7 Conclusion: Toward Biology‐Centric Laser Dermatology

Biology‐guided treatment planning must evolve beyond phenotype‐based approximations that inadequately capture the complexity of skin physiology. While established laser safety standards remain essential for operator protection, patient protection, device use, training, and administrative protocols, the present perspective highlights an additional clinical dimension: The use of biological and molecular predictors to improve individualized risk assessment before energy‐based treatments.

As dermatology enters an era increasingly shaped by precision medicine, quantitative markers such as skin barrier function, inflammatory signatures, pigmentary reactivity, oxidative stress, microbiome status, and biological age may help refine treatment planning and complication prevention. These parameters may provide insight into the skin's capacity to tolerate energy, repair injury, and remodel effectively after laser exposure.

Importantly, many of these tools remain investigational or limited to specialized settings, and further clinical validation is needed before routine implementation. Future studies should determine which biomarkers are most reproducible, clinically feasible, cost‐effective, and predictive of adverse events across diverse phototypes and patient populations.

Ultimately, biology‐centric laser dermatology should be viewed as a complementary evolution of current practice rather than a replacement for established safety frameworks. By integrating clinical expertise, conventional safety principles, objective skin assessment, and emerging molecular data, the field may move toward more personalized, predictable, and equitable energy‐based treatments.

## Author Contributions

D.H. performed the research, designed the research study, and wrote the paper. AdF, G.R.F., MR, and A.B. reviewed and edited the paper. All authors have read and approved the final manuscript.

## Funding

The authors have nothing to report.

## Ethics Statement

The authors have nothing to report.

## Conflicts of Interest

The authors declare no conflicts of interest.

## Data Availability

Data sharing not applicable to this article as no datasets were generated or analysed during the current study.
